# Role conflict, satisfaction, and performance in administrative staff at public universities: The role of optimism and sex differences

**DOI:** 10.1371/journal.pone.0321643

**Published:** 2025-04-29

**Authors:** Pedro Antonio Díaz-Fúnez, Francisco Gabriel Martín-Martín, Ana Martínez-Díaz, Carmen Salvador-Ferrer, Chiara Consiglio, Miguel Ángel Mañas-Rodríguez

**Affiliations:** 1 IPTORA Research Team, Department of Psychology, University of Almeria, Almeria, Spain; 2 Department of Psychology, Sapienza University, Roma, Italy; The World Islamic Sciences and Education University, JORDAN

## Abstract

**Background:**

This study addresses a critical gap in the literature by examining how optimism can moderate the effects of role conflict specifically among administrative staff at public universities. Role conflict is a prevalent challenge for these workers, who must navigate the demands of various groups with opposing needs, such as the university’s governing body, different professors and researchers, students, and suppliers. This situation can be a stressor that depletes their resources. Previous research has shown the negative impact of role conflict on job satisfaction and performance; however, few studies analyze this relationship in administrative employees at public universities considering their individual characteristics. One individual factor that has been shown to mitigate the negative effects of role conflict is employees’ optimism, but it is necessary to analyze whether this effect is also observed in this uniquely characterized work context and across both sexes (women/men).

**Objectives:**

The purpose of this study is to verify whether role conflict has a negative impact on job satisfaction and perceived performance among administrative employees. Additionally, it aims to investigate whether employees’ optimism moderates the negative effects of role conflict on job satisfaction and performance. Moreover, this study will explore whether the effects of role conflict and optimism vary based on the employee’s sex (women/men), considering potential differences in how these factors influence their experiences and outcomes in the workplace.

**Design and Methods:**

Data were collected from 334 employees with administrative and customer service roles at a public university (172 men and 162 women). This sample size provides sufficient statistical power to detect significant effects, allows for reliable subgroup analysis by gender, and aligns with typical sample sizes in organizational and psychological research.

**Results:**

The results confirm that role conflict has a significantly negative influence on performance through job satisfaction (mediation effect), although only in the men group. Moreover, this process is moderated by optimism (Men: IE =.0733, SE =.028, 95% CI BC from.0210 to.1296; Women: IE =.0312, SE =.046, 95% CI BC from -.0660 to.1146).

**Conclusions:**

It is concluded that role conflict negatively affects the perception of one’s own performance among administrative employees. However, while in the men group this relationship is mediated by job satisfaction and moderated by optimism, in women, role conflict directly affects performance. This difference may be due to distinct coping strategies and emotional responses to work demands between men and women, which influence how job satisfaction impacts perceived performance. These findings suggest that interventions aimed at increasing optimism and other personal resources may be particularly effective in mitigating the negative effects of role conflict, especially in men. Nonetheless, in the case of women, it is important to further investigate other potential individual factors that may moderate role conflict, as well as implement strategies that directly reduce stress sources in the workplace.

## Introduction

Universities are institutions of higher education whose aim is to disseminate and debate societal knowledge, training future professionals and researchers, and driving scientific advancement [1]. Public universities, in particular, play a vital role in cultural and academic development [2]. However, their staff face a highly demanding and complex environment, shaped by ongoing changes in technology, policies, and organizational structures [3]. Administrative and service employees in public universities are essential for ensuring the smooth operation of these institutions [4]. Unlike faculty and researchers, this group often handles diverse and sometimes conflicting demands from multiple stakeholders, leaving them particularly vulnerable to role conflict [5,6].

In this context, role conflict occurs when a position requires employees to fulfill multiple, incompatible tasks or objectives, making their simultaneous execution difficult [7]. Facing a conflict situation has proven to be costly for the resources available to employees, generating negative effects on both emotional states, such as job satisfaction, and work behaviors, such as performance [8,9]. However, few studies address these effects on administrative employees of public universities compared to other groups [10].

Optimism, defined as a general expectation of achieving positive outcomes, is a critical personal resource for managing work demands degrees [11]. Individuals with high levels of optimism interpret work demands as opportunities for personal growth, which has been shown to reduce the negative impact of these demands [12,13]. For example, several studies found that optimistic employees in high-stress environments, such as healthcare and education, were more likely to engage in positive cognitive reappraisal strategies, reframing conflicting demands as manageable challenges rather than insurmountable obstacles [14,15]. This cognitive shift helped them maintain higher levels of job satisfaction and motivation, ultimately mitigating the negative effects of role conflict on their performance. Cultural sex roles also shape optimism in the workplace, as societal expectations influence how men and women develop and utilize personal resources [16]. Research suggests that women, often expected to exhibit greater resilience and persistence, may rely more on optimism as a coping mechanism, while men may adopt different strategies [17–19]. These differences underscore the need to consider sex as a factor in understanding the effectiveness of optimism in addressing role conflict [20,21].

This research aims to examine the relationship between role conflict, job satisfaction, and perceived performance among administrative and service employees in public universities. Specifically, it investigates how varying levels of optimism interact with role conflict to influence job satisfaction and perceived performance. Additionally, the study explores whether these relationships differ based on employees’ sex.

## Theoretical framework

The constant changes faced by employees at public universities have increased the perception of demands such as role conflict [4]. Role conflict arises when an employee has to perform incompatible tasks, when the jobs and responsibilities of one task overlap with those of others, or when contradictory requests occur [22]. However, the perception of role conflict has a significant subjective component. This perception also arises when a worker believes that the tasks, they must perform are unethical or not part of their functions [23]. Additionally, conflict intensifies when there are objectives that contradict bureaucratic procedures in public administrations [24,25]. In public universities, role conflict can manifest in several ways. For example, administrative staff often find themselves caught between the conflicting demands of faculty, students, and external stakeholders. They may need to manage competing priorities, such as meeting strict regulatory deadlines, addressing urgent students concerns, fulfilling external audit requirements, and ensuring the smooth operation of daily academic activities, among other [26].

One theory that explains the negative effect of role conflict on employee well-being is the Conservation of Resources (COR) theory [27,28]. To manage tasks or objectives that generate conflict, individuals must make additional use of their resources (e.g., negotiating with colleagues, duplicating work, making an extra effort) or reorient available resources to mitigate this loss. This reduces their normal working capacity and generates a perception of discomfort [29–31].

Derived from the discomfort associated with resource loss, one of the most sensitive aspects in the face of role conflict is the perception of one’s own performance [32]. This is defined as the employee’s opinion about the work results achieved over a specific period [33]. When this perception is positive, it becomes a factor associated with objective productivity and the achievement of organizational goals [34]. However, it is greatly affected when the employee must devote available resources to incompatible objectives or tasks, characteristic of conflict situations [7]. In these circumstances, workers are forced to make decisions and act in a manner that is inconsistent and ineffective, perceiving a reduction in their productive capacity [35,36]. Despite solid findings on this negative influence, few studies have delved into the existence of mediating and moderating variables that could intervene in this influence process [37].

An emotional state that has been shown to have a specific influence on the perception of one’s own performance in the workplace context is job satisfaction [38]. This is defined as the positive emotional experience an employee feels towards their job [39]. According to the Conservation of Resources (COR) theory [27,28], when employees perceive that their resources are being depleted due to additional and contradictory demands, their job satisfaction decreases. This decline occurs because the extra effort required to manage the conflict depletes personal resources, leading to lower engagement, energy, and enthusiasm toward work [39,40].

Studies in public universities have specifically linked excessive bureaucratic demands and unclear role expectations to diminished job satisfaction among administrative staff. For example, recent research in higher education institutions has found that administrators who frequently face conflicting instructions from multiple departments report lower levels of motivation and engagement, which directly impacts their job satisfaction [41,42]. Furthermore, the rigid structures typical of public institutions can limit employees’ autonomy, exacerbating dissatisfaction when they are unable to make independent decisions to resolve conflicts effectively [4,9].

Most studies have focused on private sector organizations, which have very different characteristics from administrative staff in public universities, though it is hypothesized that the effects will follow a similar trend [43,44]. However, given the increasing administrative burden and conflicting responsibilities in public universities, the negative impact of role conflict on job satisfaction may be even more pronounced in this sector. Therefore, the following hypothesis is proposed:

### H1: Role conflict will have a negative effect on job satisfaction among administrative employees at public universities

Research has demonstrated that role conflict significantly influences job satisfaction, which in turn affects employees’ perceived performance [36,37]. Several studies in public universities have shown that employees experiencing high levels of role conflict report lower job satisfaction, which negatively impacts their work engagement and efficiency [4,35,45]. Job satisfaction acts as a mediating variable in the relationship between work stress and perceived performance [32]. For instance, research on administrative staff in public universities has found that bureaucratic constraints, conflicting responsibilities, and lack of autonomy reduce job satisfaction, which subsequently leads to lower self-assessment of performance [46]. These findings indicate that the greater the impact of a demand on an employee’s job satisfaction, the greater the negative effect on their perceived performance. In this context, the Conservation of Resources (COR) theory [27,28] posits that resource loss is a motivational process affecting job satisfaction and, consequently, performance. When employees perceive their resources are depleting due to additional and contradictory demands, their job satisfaction decreases, leading to a lower perception of their own performance [47].

However, this mediating effect may vary depending on the type of administrative tasks performed and the organizational structure of the university. For example, employees involved in student services may experience higher emotional demands due to direct interaction with students, while those working in financial or regulatory compliance roles may face increased bureaucratic constraints. These differences could amplify or mitigate the impact of role conflict on job satisfaction and, consequently, on perceived performance. Therefore, we propose the following hypothesis:

### H2: The direct effect of role conflict on perceived performance will be mediated by job satisfaction among public university employees

In the same way, the Conservation of Resources (COR) model [28] emphasizes the importance of personal resources as determinants in evaluating demands. These resources are characterized by self-evaluations regarding one’s capacity to control and influence the environment [27,48]. One of the most studied personal resources in this context is optimism [21]. Optimistic serves as a psychological resource that helps employees reframe stressful situations, reducing the perceived threat of role conflict [19,20]. Optimistic individuals are more likely to engage in adaptive coping strategies, such as cognitive reappraisal and problem-focused-coping, which allow them to manage conflicting demands more effectively [49]. By interpreting challenges as opportunities rather than threats, optimism helps mitigate the negative emotional response to role conflict, preserving job satisfaction [50]. Furthermore, optimism fosters persistence and proactive behavior, enabling employees to sustain motivation and performance even in the presence of conflicting demands [11].

In line with COR theory, optimism acts as a protective factor against resource depletion, as optimistic individuals are more likely to recover lost resources or maintain engagement despite difficulties [14,51,52]. This suggest that optimism does not eliminate the negative effects of role conflict but buffers its impact by maintaining higher levels of job satisfaction and motivation, which ultimately supports perceived performance. Therefore, we propose the following hypothesis:

### H3: Optimism will have a moderated mediation effect on the influence of role conflict on perceived performance through job satisfaction among public university employees

An understudied aspect of the work context is the difference in responses between men and women to demanding situations [53]. Although women show greater sensitivity to demands, they tend to possess a greater number of personal resources, such as optimism [17]. These differences are also reflected in the coping strategies they employ when facing role conflict. Research suggests that women are more likely to use emotion-focused coping strategies, such as seeking social support, engaging in collaborative problem solving, and reframing stressors in a more positive light [54,55]. In contrast, men tend to adopt more problem-focused or avoidance strategies, prioritizing task completion and individual problem-solving over emotional regulation.

For example, in the context of public universities, a women administrative employee experiencing role conflict due to conflicting demands from faculty and students may proactively seek support from colleagues or supervisors, engaging in discussions to find a collective solution [56]. On the other hand, a male administrative employee in the same situation might attempt to handle the issue independently, focusing on processual efficiency and problem resolution without necessarily addressing the emotional toll [57]. Therefore, this study considers differences in the perception and response to work demands according to the sex of employees. For this reason, the following hypothesis is proposed:

### H4: The moderating effect of optimism on the influence of role conflict on job satisfaction and in-role performance will differ depending on the sex of public university employees

**Fig 1:**
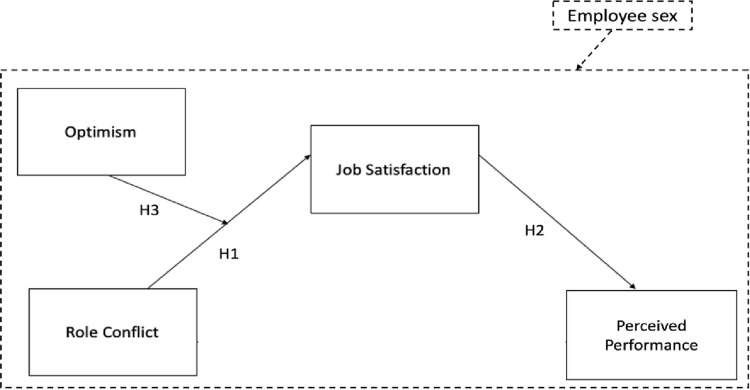
Research model.

The objective of this study is to delve into the effects of role conflict, a common demand among employees with administrative tasks in public universities. Administrative staff play a crucial role in ensuring the efficient operation of universities, acting as the backbone of institutional management [2]. Unlike faculty members, whose primary focus is teaching and research, administrative employees must navigate a complex landscape of bureaucratic procedures, regulatory compliance, and service-oriented responsibilities [3,4]. Their work requires balancing the often-conflicting demands of faculty, students, external agencies, and university leadership, making them particularly vulnerable to role conflict [5]. The proposed hypotheses suggest that this demand has a negative effect on employees’ perceived performance through job satisfaction and that optimism acts as a personal resource moderating this influence. Furthermore, this study considers the possible differences in this process according to the sex of the employee. Fig 1 illustrates the research model that guides this study.

## Materials and methods

### Study desing

This study employed a cross-sectional design to examine the impact of role conflict on perceived performance, mediated by job satisfaction and moderated by optimism, among administrative employees in a public university.

### Setting

Data collection took place in a Spanish public university between September 10^th^, 2018, and December 30^th^, 2018. Participants completed the survey during their working hours through an online platform. The University Ethics Committee approved the study beforehand (UALBIO2018/027).

### Participants

The final sample for this study consisted of 334 administrative employees from a public university, of whom 172 were men (51.6%) and 162 were women (48.4%). The participants’ ages ranged from less than 36 years to over 55 years, with the majority being between 46 and 55 years old (61.7% of men and 66.5% of women). Regarding educational background, most participants held an undergraduate degree (56.0% of men and 54.9% of women), followed by those with a high school diploma. A small percentage had advanced studies or a doctorate. The detailed demographic breakdown of the sample is presented in Table 1.

**Table 1:** Demographic Data of the Sample.

	Men (n = 172)	Women (n = 162)
**Sex**	51.6%	48.4%
**Age**		
Less than 36 years	1.7%	1.8%
36 y 45 years	15.1%	10.9%
46 y 55 years	61.7%	66.5%
More than 55 years	18.4%	20.7%
**Educational Level**		
Primary Education	4.6%	1.8%
Secondary Education	6.9%	7.3%
High School	21.7%	24.4%
Undergraduate	56.0%	54.9%
Advanced Studies	9.7%	10.4%
Doctorate	1.1%	1.2%

### Measures

#### Role conflict.

This dimension was measured using the Role Stress Questionnaire [58], adapted by [59]. It consists of 8 items (e.g., “I receive incompatible demands from two or more people at work”). Responses were rated on a 5-point Likert scale, ranging from 1 (strongly disagree) to 5 (strongly agree). The Cronbach’s alpha coefficient for this scale was.90. This questionnaire has been previously validated in Spanish-speaking samples, ensuring its cultural applicability and reliability in this context.

#### Optimism.

This dimension was measured using the Spanish adaptation [60] of the Psychological Capital Questionnaire (CPQ-24) by [61]. It consists of 3 items (e.g., “I always look on the bright side of things related to my job”). All items were rated on a 6-point Likert scale, ranging from 1 (strongly disagree) to 6 (strongly agree). The Cronbach’s alpha coefficient for this scale was.85. Previous studies have confirmed the validity and reliability of this Spanish adaptation in similar cultural contexts.

#### Job satisfaction.

Job satisfaction was measured using the Job Satisfaction Scale (CSLPS-EAP/33) by [62], adapted to a specific sample of public administration employees, according to the Spanish version of [63].It consists of 33 items (e.g., “Indicate the degree of satisfaction or dissatisfaction you feel with the rules and norms of operation in your work team”). Responses were rated on a 7-point Likert scale, ranging from 1 (very dissatisfied) to 7 (very satisfied). The Cronbach’s alpha coefficient for this scale was.82. This adaptation has been validated in Spanish-speaking work environments, supporting its relevance for this study.

#### Performance.

This dimension was measured using the Spanish adaptation of the Goodman and Svyantek scale [64] by [65]. It consists of 3 items (e.g., “I achieve my work objectives”). Items were rated on a 6-point Likert scale, ranging from 1 (strongly disagree) to 7 (strongly agree). The Cronbach’s alpha coefficient for this scale was.94. The Spanish version of this scale has demonstrated strong psychometric properties in previous research, reinforcing its reliability for this study.

### Procedure

The research team contacted the administration’s management and explained the project’s purpose. Once they agreed to collaborate, the employees from each department were informed by the management about the study’s relevance, aiming to secure participation and ensure confidentiality and anonymity. Participants completed the questionnaires online during their working hours. If they had any questions, members of the research team were available to assist.

### Data analysis

All data analysis were conducted using SPSS 29. After calculating descriptive statistics, Cronbach’s alpha, and zero-order correlations among all constructs, mediation and moderation analyses were performed. Initially, the independent variable (role conflict), the mediator (job satisfaction), and the moderator (optimism) were mean centered to avoid potential multicollinearity issues [66].

To further assess multicollinearity, Variance Inflation Factors (VIFs) were calculated for all predictor variables. All VIF values were below the commonly accepted threshold of 10, indicating that multicollinearity was not a concern in the analyses.

Separate mediation and moderation analyses were performed for men and women to estimate direct and indirect influences using the nonparametric bootstrapping procedure from the PROCESS macro [67]. Following Hayes’ recommendation, a stepwise mediation analysis of the influence of role conflict on performance perception was first conducted (Model 4 in PROCESS), with job satisfaction as the mediator and optimism as the moderator (Model 7 in PROCESS).

Indirect and conditional influences were considered significant if the 95% bootstrapped confidence intervals (BC) based on 10,000 samples did not include zero. Effect sizes for the mediation were calculated using the completely standardized indirect effect (abcs) [68], providing 95% BC confidence intervals. This effect size measure is based on the product of betas for the paths a and b and can be interpreted as the expected change in the dependent variable (i.e., performance perception) per unit change in the predictor variable (i.e., role conflict) that occurs indirectly through the mediator (i.e., overall satisfaction). Finally, the Johnson-Neyman technique [69] was used to derive the moderator value (i.e., optimism) at which the influence of the predictor variable (i.e., role conflict) changes from statistically significant to non-significant at an alpha level of.05.

## Results

Table 2 presents the descriptive statistics, internal consistencies, and correlations for each variable for men and women in the sample. For both sexes, the mean scores for job satisfaction, optimism, and performance perception were above the midpoint of their respective measurement scales (midpoints: 4 for job satisfaction and perceived performance and 3.5 for optimism). In contrast, the mean score for role conflict was below the midpoint of the measurement scale for this variable (midpoint of role conflict: 3). The internal consistencies of the scales ranged from.70 (optimism in women) to.92 (performance perception in men) and were similar to previous findings using these instruments. All constructs were significantly correlated with each other, with negative relationships for role conflict and positive relationships among the rest of the variables. There were no significant differences in mean scores for the variables based on employees’ sex.

**Table 2:** Descriptive Statistics, Internal Consistencies, and Correlations for Men and Women.

	M	SD	α	2	3	4
**Men**						
1. Role Conflict	2.56	.85	.85	−.420***	−.337***	−.317***
2. Job Satisfaction	5.35	1.4	.90		.580***	.455***
3. Optimism	4.32	1.11	.75			.452***
4. Performance Perception	4.88	1.02	.92			
**Women**						
1. Role Conflict	2.69	.95	.88	−.389***	−.333***	−.389***
2. Job Satisfaction	5.37	1.42	.89		.598***	.595***
3. Optimism	4.47	1.09	.70			.538***
4. Performance Perception	4.83	1.08	.91			

**Note:** *M = Mean; SD = Standard Deviation; α = Cronbach’s Alpha*

***p ≤.001,

**p ≤.01,

*p ≤.05

### Regression models in men

Table 3 reports the results of the models tested in the mediation analysis for men. In Model 1, role conflict was a significant and negative predictor of the mediator (job satisfaction). According to Model 2, the total influence of role conflict on performance perception was also significant and negative (β = -.381, SE =.088, p <.001). Model 3 shows that the coefficient for role conflict decreased from β = -.381 to β = -.164 when all variables were included in the regression analysis, becoming non-significant (p =.078).

**Table 3:** Results of regression analyses examining the mediation model of the influence of role conflict (X) on performance perception (Y) through job satisfaction (M) in men.

	Coefficient	SE	p
**Model 1 (Job Satisfaction)**			
X (Role Conflict)	−.689	.113	< .001
Constant	7.110	.305	< .001
R^2^= .176 F = 36.985, P ≤ .001			
**Model 2 (Performance Perception)**			
X (Role Conflict)	−.381	.088	< .001
Constant	5.859	.237	< .001
R^2^ = .101 F = 18.830, P ≤.001			
**Model 3 (Performance Perception)**			
X (Role Conflict)	−.164	.093	.078
M (Job Satisfaction)	.288	.057	< .001
Constant	3.753	.470	< .001
R^2^ = .221 F = 23.738, P ≤ .001			

**Note:** *SE = Standard Error; R² = Coefficient of Determination; F = F-statistic; p = p-value.*

Table 4 presents the indirect effect of role conflict on performance perception through job satisfaction in men. The results show a significant mediation, with a total Indirect Effect of β =.0733 (SE =.028, 95% BC CI [.0210,.1296]) and an ab_cs_ effect size = -.191.

**Table 4:** Indirect effect of role conflict (X) on performance perception (Y) through job satisfaction (M) in men.

	Coefficient	SE	Bootstrapping	
			BC 95% CI	
			Lower	Upper
Indirect effect	.0733	.028	.0210	.1296

**Note:** *SE = Standard Error; BC CI = Bias-Corrected Confidence Interval.*

Table 5 presents the results of the moderation analysis of optimism in men. These show that the effect of role conflict on job satisfaction is moderated by the level of optimism, changing from having a negative and significant coefficient (β=-1.606, p<.001) to the coefficient of positive and significant interaction (X*W:.254, p<.05). Specifically, the Johnson-Neyman technique indicated that the negative conditional influence of role conflict on job satisfaction decreases as optimism score increases, ceasing to have a significant impact for optimism scores higher than 5.35.

**Table 5:** Results of the moderation analysis of optimism on the impact of role conflict on job satisfaction in men, based on the Johnson-Neyman technique.

Antecedents	Coefficient	SE	p
X (Role Conflict)	-1.606	.379	.000
W (Optimism)	-.086	.235	n.s.
X*W	.254	.082	.002
Constant	7.130	1.133	.000
R^2^ = .452 F = 45.643, P=.000			
Johnson-Neyman technique			
Optimism scores	Coefficient	SE	T
1.00	−1.352	.300	−4.493***
1.25	−1.288	.282	−4.573***
1.50	−1.225	.263	−4.662***
1.75	−1.161	.244	−4.760***
2.00	−1.098	.225	−4.869***
2.25	−1.034	.207	−4.987***
2.50	−.971	.189	−5.115***
2.75	−.907	.173	−5.251***
3.00	−.843	.157	−5.388***
3.25	−.779	.141	−5.513***
3.50	−.716	.128	−5.602***
3.75	−.653	.116	−5.612***
4.00	−.589	.107	−5.485***
4.25	−.526	.102	−5.157***
4.50	−.462	.100	−4.605***
4.75	−.399	.103	−3.873***
5.00	−.335	.109	−3.065**
5.25	−.271	.119	−2.283*
5.35	−.244	.124	−1.974*
5.50	−.208	.131	−1.587
5.75	−.144	.145	−.996
6.00	−.081	.160	−.504

***Note:*** *SE = Standard Error; R² = Coefficient of Determination; F = F-statistic; T = T-statistic; p = p-value.*

***p ≤.001;

**p ≤.01;

*p ≤.05

### Results in women

Table 6 reports the results of the mediation models in the women group. In Model 1, role conflict was a significant negative predictor of the mediator (job satisfaction) with a coefficient of β=-.585 (p<.001). According to Model 2, the total influence of role conflict on perceived performance was also negative and significant in this group (β= -.449, SE =.089, p <.001). Model 3 shows that the coefficient of role conflict decreased from β=-.449 to β=-.192 when all variables were included in the regression analysis.

**Table 6:** Results of the regression analyses examining the mediation model of the influence of role conflict (X) on perceived performance (Y) through job satisfaction (M) in women.

	Coefficient	SE	p
**Model 1 (Job Satisfaction)**			
X (Role Conflict)	−.585	.109	< .001
Constant	6.943	.310	< .001
R^2^ = .152 F = 28.939, P ≤ .001			
**Model 2 (In-role Performance)**			
X (Role Conflict)	−.449	.089	< .001
Constant	6.015	.250	< .001
R^2^ = .152 F = 25.566, P ≤.001			
**Model 3 (In-role Performance)**			
X (Role Conflict)	−.192	.084	<.05
M (Job Satisfaction)	.39	.054	< .001
Constant	3.265	.440	< .001
R^2^ = .377 F = 42.987, P ≤ .001			

*Note: SE = Standard Error; R² = Coefficient of Determination; F = F-statistic; T = T-statistic; p = p-value.*

Table 7 presents the indirect effect of role conflict on perceived performance through job satisfaction. The results show mediation with reduction, but not significant, with a total Indirect Effect of β=.0312 (SE =.046, 95% BC CI [-.0660,.1146]) and an effect size of ab_cs_ = -.232.

**Table 7:** Indirect effect of role conflict (X) on perceived performance (Y) through job satisfaction (M) in women.

	Coefficient	SE	Bootstrapping	
			BC 95% CI	
			Lower	Upper
Indirect Effect	.0312	.046	-.0660	.1146

*Note: SE = Standard Error; CI = Bias-Corrected Confidence Interval.*

Table 8 presents the results of the moderation analysis of optimism in the group of women. The results show that the effect of role conflict on job satisfaction was not moderated by optimism in the group of women (β=.080, SE=.084, p=n.s.), despite the significant direct effect of optimism on job satisfaction reported (β=.496, SE=.238, p<.05). The Johnson-Neyman technique does not present results due to the lack of significant conditional influence of optimism on the effect of role conflict on job satisfaction.

**Table 8:** Results of the moderation analysis of optimism on the impact of role conflict on job satisfaction in women.

Background	Coefficient	SE	p
X (Role Conflict)	-0.723	.380	n.s.
W (Optimism)	.496	.238	<.05
X*W	.080	.084	n.s.
Constant	4.108	1.144	.000
R^2^ = .433 F = 35.940, P = .000			

*Note: SE = Standard Error; R² = Coefficient of Determination; F = F-statistic; T = T-statistic; p = p-value.*

## Discussion

This study confirms the negative impact of role conflict on job satisfaction and perceived performance among administrative employees at public universities, in line with the Conservation of Resources (COR) theory [40,41]. The findings show that while this negative influence is present for both men and women, the mediation process through job satisfaction only appears significant in men. In women, job satisfaction does not act as a mediator between role conflict and performance perception, suggesting that different pathways might be at play depending on sex.

The role of optimism as a personal resource also highlights an interesting difference. For men, optimism moderates the relationship between role conflict and job satisfaction, buffering its negative effects and preserving perceived performance. This aligns with previous studies showing that optimism promotes resilience and adaptive coping strategies [23,24,57,59]. However, in women, optimism improves job satisfaction but does not moderate the influence of role conflict on performance, pointing to the potential importance of other protective factors such as social support or self-efficacy [70,71].

These findings underline the need for tailored interventions. For men, enhancing optimism may reduce the negative impact of role conflict, improving job satisfaction and performance. For women, interventions should focus on alternative resources beyond optimism to address role conflict’s negative effects.

### Theoretical implications

The results found in this work allow us to propose four theoretical implications. The first confirms the direct negative effect of role conflict on job satisfaction and performance perception among administrative workers at a public university. These results support the tenets of the Conservation of Resources Theory [27,28] and align with previous findings in this area [35,36], and [70]. The results of this study occur in the context of a public university, reinforcing the idea of the universality of the negative effects of role conflict as a demand, regardless of the characteristics of the organization where they occur [1].

A second theoretical contribution of this study is the confirmation of different pathways through which role conflict can affect performance perception. In the case of men employees working on administrative tasks at the public university, the results prove that the process of influence of role conflict on performance perception is mediated by job satisfaction. This result aligns with the previous findings that advocate for the mediating effect of this variable. However, in the case of women, job satisfaction has not shown this mediating effect. This allows us to affirm that the negative influence of role conflict on perceived performance does not follow a single process but follows different paths depending on the group affected. This proposes a new line of research that delves into studying the different ways through which role conflict affects performance perception in each group despite its direct negative influence in all cases.

A third theoretical contribution of this study derives from the partial confirmation of the moderated mediation effect of optimism on the influence of role conflict on performance perception through job satisfaction. The Conservation of Resources Theory [28] includes optimism as a key personal resource in situations with high levels of demands. In line with studies in different groups [11,51,14], the findings of this study show that public university administrative employees with higher levels of optimism apply a positive approach to work, reducing the negative effect of role conflict on satisfaction and performance perception. However, the results indicate that this effect appears only in the group of men. This is because the impact of role conflict on performance perception is not mediated by job satisfaction in the case of women. One possible explanation for this finding is that men and women may rely on different personal resources to cope with role conflict. While optimism serves as a protective factor for men by mitigating its negative effects through job satisfaction, women might employ alternative coping strategies, such as resilience, social support, or self-efficacy. These factors could play a more significant role in buffering the impact of role conflict on performance perception, which would explain why optimism does not exert the same mediating effect in this group. Future research should explore these alternative mechanisms to provide a more comprehensive understanding of how personal resources interact with job demands in different gender groups.

The fourth theoretical implication refers to the differential effect in the moderated mediation of optimism according to the sex of the employees. Previous studies have suggested that different conditions in the workplace can affect the relationships between job demands and resources [71]. The findings have confirmed that the cultural roles associated with each sex enable the existence of different sources of personal resources and differences in how they are used [16,17,53]. The results of this study indicate that the influence of role conflict on performance occurs through different processes in men and women, proposing this reason as a possible cause of the differences found in previous studies according to sex. It is possible that the most important process mediating the impact on performance is not motivational (through satisfaction) but rather through conflict. At the same time, the most important individual variables for women could be others, suggesting that, although optimism is a valuable resource, some research has shown that factors such as resilience, social support, or self-efficacy can play a more crucial role in mitigating the negative impact of role conflict [72,73].

### Practical implications

The findings of this study present several practical implications for public universities, highlight the importance of addressing role conflict and promoting personal resources to enhance employees well-being and performance.

First, these results emphasize the need to prevent role conflict among administrative staff due to its negative effects on job satisfaction and performance perception. Public universities should implement structured interventions, such as conflict management training and resilience development programs, to help employee recognize and effectively manage conflicting demands. Providing employees with these tools could reduce the adverse impact of role conflict and improve their overall work experience. Second, since men and women respond differently to role conflict, it is essential to tailor interventions according to the employee’s sex. For men administrative employees, strategies that promote optimism can be highly effect. For male administrative employees, strategies that promote optimism can be highly effective in reducing the negative impact of role conflict on job satisfaction and performance. Public universities could offer positive psychology interventions, cognitive-behavioral training, and coaching sessions to strengthen optimism and proactive coping strategies.

In contrast, for female employees, optimism alone may not be sufficient to mitigate the adverse effects of role conflict. Therefore, universities should explore additional resources such as resilience training, social support programs, and mentoring systems to strengthen other protective factors. Creating peer-support networks within administrative teams may also help women build stronger emotional and practical support systems, reducing the perceived impact of conflicting demands.

Finally, it is recommended that public universities regularly assess employee well-being and organizational demands through periodic surveys and feedback mechanisms. Continuous monitoring would enable the early identification of high levels of role conflict and allow institutions to implement targeted interventions in a timely manner. This strategy would also provide data to evaluate the long-term effectiveness of these interventions, ensuring that policies remain adaptive and relevant to employees’ evolving needs.

### Limitations and future research

Despite the results achieved in this study, it is important to note some limitations that should be considered to expand knowledge on the objective of the present study. Firstly, one limitation lies in the cross-sectional nature of the study design. This design makes it difficult to detect temporal fluctuations in the influence of the context, which could impact the relationships between the analyzed variables. A longitudinal study would allow testing of the mediation process over time, for example, through a cross-lagged model. Additionally, future research could employ mixed-method approaches, combining qualitative and quantitative data, to provide a more nuanced understanding of role conflict over time.

The second limitation refers to the data collection methodology. In this case, self-report measures were used from the same individuals in a single application. Although this approach is commonly used in psychology, future research could consider including other types of analysis with a higher qualitative index to explain the quality of emotional and instrumental relationships among employees (e.g., employee interviews), observation of the organization, and collection of objective organizational data (e.g., performance evaluated by the manager or other productivity and performance indicators) to ensure bias reduction [74]. Organizational behaviors beyond performance, such as absenteeism or work errors, could also be collected. Moreover, incorporating multisource data, such as peer evaluations or third-party assessments, could help mitigate the limitations associated with self-reported measures and reduce potential biases.

Based on the results, which have partially confirmed the hypotheses, future studies could expand the possible mediation and moderation effects studied to investigate the different processes between men and women. According to Hobfoll’s model [28], resource losses due to job demands affect not only motivation but also stress. Therefore, future studies could evaluate the mediation of burnout instead of job satisfaction. Additionally, they could explore the potential protective role of other individual variables that may be important for coping with role conflict, such as resilience or self-efficacy, in men and women.

Finally, although this research provides relevant information on how public organizations are affected by role conflict in Spain, this study was conducted specifically within the context of a public university. This could lead to a coverage bias and calls for caution when generalizing these findings to other types of organizations. In future studies, an interesting aspect would be to include employees from different entities, both public and private.

### Conclusion

In conclusion, this study contributes to corroborating the universal negative effect of role conflict as a demand, showing its negative influence on job satisfaction and performance perception among public university administrative employees. At the same time, it has shown that this influence process presents differences depending on the sex of the public employees, conditioning the beneficial effect of a personal resource like optimism in this context. This makes the effectiveness of intervention processes designed to reduce role conflict by increasing personal resources in public administration need to be adapted to the group where they will be implemented. Furthermore, while this study was conducted in a public university setting, the findings could be relevant to other sectors facing similar organizational challenges. Future research could explore the extent to which these results apply to different work environments, such as private sector organizations or other public institutions, to enhance the generalizability of these insights and their practical implications.
